# Editorial and Miscellaneous

**Published:** 1861-12

**Authors:** 


### EDITORIAL.

*The Reform School.*—Turning a leaf in the chronicles of medical reform, we find full tickets—matriculation, dissecting, lecture and hospital inclusive, amounting to *sixty-six dollars*, offered al head-quarters for *twenty-five dollars*—a discount of only *forty-one dollars* for the promotion of the great cause of professional elevation! Cheap at half the money, but, alas, for prospects of speedy reformation, the "beggarly account of empty boxes" poorly represents the richness of the promise of the tympanitic matriculation list. The stock is heavy on the market, and buyers are wary, although the "bulls" are doing their best to inflatethe "fancy." The latest project for "a Royal Road to Knowledge" threatens incontinent collapse.

*Swinging in Consumption.*—The *Courrier des Etats-Unis* states that a physician in Constantinople has just proved that swinging is a useful remedy in consumption, and calls upon the profession to test its peculiar effects. The Romans used swinging bedsin various disorders, and even Celsus advised them in several chronic diseases. There is an element of truth in the proposition. Gentle exercise, involving use of all the muscles in the body, is among the very best of remedies for phthisis, because it is one of the best promovents of digestion, tissue metamorphosis, and assimilation. There is no greater folly than shutting up the phthisical patient and condemning him to inaction. Open-air pleasurable exercise, generous food and drinks, freedom from mental anxieties, and a steady determination not to succumb to the disease, will cure more cases than cod-liver oil, cohosh or even chlorate of potash. Tubercular phthisis is no more essentially a disease of the lungs, than is gout a disease of the toe. Yet expectorants and inhalations retain their ascendancy in many minds—antimony and digitalis have their advocates, and scores of specifics, or, in more modest terms, " medicines good for consumption," are thrust in the face of the profession with an obtuse perseverance worthy of the dark ages.

---

*Mercurials in Remittent Fever.*—A correspondent of the *Lancet and Observer* states, as the result of sixteen years practice, that he finds it unnecessary to use mercurials of any sort in the treatment of bilious remittent. More than this, he argues that they are theoretically improper, because they tend to disintegrate nitrogenized tissues, whereas in remittent the demand is for elimination of carbonaceous material alone—the azotized tissues not requiring increase of tissue metamorphosis. The theorizing he indulges in, which of course can be easily shown wholly untenable in its grounds, may be passed over for his practical conclusion, that mercurials are useless or positively injurious in the treatment of this variety of endemic disease.

We suspect the vast majority of practitioners in the West aud South will disagree with the conclusions of the correspondent. Remittents may be treated successfully often, without mercurials. The writer has often done this, but, from long experience and close observation, he has found that a judicious use of these potent agents materially facilitates the rapidity and perfection of the cure. Any cathartic which produces dark tarry discharges, not serous but of some consistency, will immensely assist the beneficent impression of the Quinine. Cathartics which do not yield this product aggravate the disease. It is folly to wait for any sort of cathartics or alteratives to procure intermissions of a decided character before employing the antiperiodic; yet, if moderate catharsis does bring on such intermission, the effect of the Quina is more pleasantly secured.

The mercurial cathartic, more certainly than any other, will cause the dark stools rich in carbon. Salivation in bilious fever, (or any other fever or inflammation,) is a poisonous effect of the mineral, never, of course, to be tolerated here or elsewhere, but we have yet to find the agent which has the eliminating or depurant energy of mercury, however that may be explained. It is unnecessary to go from the one extreme to the other. Because Dr. E.'s practice, based upon calomel as the leading antiphlogistic, alterative, equallizer, etc., etc., is deadly, it is no reason to a philosophic mind that Dr. F. should scout the agent utterly. Because Dr. E. is wielding this weapon indiscriminately in the midnight darkness of his own notions, it is no sort of excuse for the homœopathist or tender-footed "regular" to forego its unequalled powers for good. The very energy and potency of the thing is what commends it to judicious employment. We repeat, the mercurial may be dispensed with in the treatment of remittent, for its effect may be approximated, with greater or less perfection, by various other agents or combinations. So may even quina be dispensed with, and its effect imitated by other antiperiodics. The prejudice against quina among the ignorant, is almost as great as that against calomel. Shall it therefore be discarded? No reasonable practitioner will admit it for an instant. The profession ought not to come down to the level of vulgar prejudices, but steadily to educate the people up to the higher plane of correct thinking.

The superiority of man over the animate creation, as has been well said, consists mainly in his power to control the physical forces of nature, and render them subservient to his own purposes, and thus the superiority of the true physician is manifested by his reducing to his control those powers or agencies, whatever their name, nature or derivation, which are capable of producing desired changes in the material constitution of the body. There is no force, however potent or apparently unmanageable but what may, by simply rubbing the Aladdin's Lamp of Knowledge, be made conducive to man's advantage. Profound theologians have proved that the very devil himself has his uses—then why not calomel?

*Eclampsia and Uræmia.*—Practical men must have noticed that the exceptions are few where in the human body apparently identical effects may not be produced by the most diverse causes. We recall the attention of those who have arrived *per saltem* at the conclusion that eclampsia is solely caused by uræmia, to the demonstrable fact that other causes may beget it. Convulsions may result from either excentric or centric causes. The centric disorder may be very various, and the excentric causes as infinite as the play of nerve conduction. The phenomena of puerperal convulsions, or mania even, may occur from a variety of causes aside from uræmia, and the inter-paroxysmal treatment required must therefore be modified according to the exigencies of each particular case.

---

*Atrocious remark of an Army Surgeon.*—A friend of ours, connected with the army as a Surgeon—since the war commenced, whilst suffering under the tenesmus of camp dysentery insisted, to the intense disgust of his classical attendant, that he was—*omne rectum.*

Such freedom with the dead languages is fundamentally reprehensible, and should be sternly rebuked.

---

*Arsenic Smoking in Asthma.*—The Chinese practice of smoking arsenic in asthma, has a confirmation as to its good effects in a case reported by F. G. Julius, M. D., in the *London Lancet.* A French lady, subject to spasmodic asthma for twenty-five years, and who had exhausted all the usual methods commenced smoking a fourth of a grain four times daily in a cigarette, continuing fourteen days with the greatest benefit to her breathing and general health. She subsequently increased the amount to upwards of three grains. The physician notes the important fact that she does not inhale the fumes and blow them out again, but when her mouth is full, she swallows the smoke. The only ill effects she has ever experienced are swelling of eyelids, and when she first commenced, slight pricking pains in the stomach. She considers herself cured. From being in a state of constant breathlessness and suffering, unable to lie down or make the slightest exertion, she is now able to go about like other persons, and is rarely threatened with an attack oftener than once in three or fourth months, and that is at once stopped by smoking arsenic with a very small quantity of belladonna or stramonium in the dose mentioned. She now uses, instead of a cigarette, a small red pipe about five inches long.

There is probably no remarkable advantage gained by smoking the arsenic over the other methods of exhibition. The Fowler's solution has long been a favorite alterative in asthma in the West. The writer knows of one case where an asthmatic has become a confirmed "arsenic eater," as the result of a prescription made by him for asthma some ten years since. The paroxysms which were then almost daily and distressingly severe, now occur only at intervals of weeks or months, and are of little severity. Discontinuance of arsenic, however, is about infallibly followed by a paroxysm. The amount taken at a dose never exceeds a fourth or half of a grain, and is very rarely used more than once or twice in three days. The general health of the individual is good, but he is not remarkably robust, although the complexion is clear and ruddy.

The remarkable powers of arsenic as a profoundly acting alterative in various neuroses, deserve more attention from the profession than they have yet received.

Asthma, like other spasmodic diseases, may result from local irritants inhaled, or carried through the blood to the contractile tissue of the smaller bronchial tubes, or again by so called reflex action, i. e. where the local changes exciting excessive contraction are induced by the conducting nervous fibre. In each case involving similar changes in the part, but requiring ever, for appropriate treatment, reference to the real cause, immediately or remotely acting. The inhalation of direct irritants is an obvious cause; digestive, hepatic, renal, cutaneous, cardiac and cerebral, respectively more or less obscure, but capable of diagnosis, and still there will remain a number of cases referrible only to hereditary, idiosyncratic, or, as many believe, wholly unaccountable influences. The number of these latter, we believe, is much less than usually supposed—a more careful scrutiny would clear up the obscurity now thought to surround the cause. It is almost safe to assume that central ganglionic change of structure, either as wrought by inflammation or other modified nutrition, will abundantly account for the balance. The well known character of the agents of use in empirical treatment of asthma, of chorea, epilepsy, neuralgia, etc., etc., indicate a community of action in that they restore the normal nutrition in the central ganglionic masses, particularly of the so called great sympathetic. Many of the phenomena of dyspepsia are referrible to similar central disease. Of course the time has gone by for scientific medical men to look upon functional disease, irrespective of tissue change, as having any existence, save in the fancies of theorists. Disease of the nervous tissue, whether ganglionic or conducting, requires as positive treatment as that of the muscular or glandular parts. The *modus operandi* of arsenic in curing asthma, is identical with that in its curing of ague, chorea, epilepsy and certain diseases of the skin. It restores the molecular integrity of the central ganglia, upon which the parts involved depend for the perfection of their nutrition and consequent perfection in functional operation. All the advances of modern physiology and pathology are slowly but surely infixing this cardinal truth that all disease is the result of modified nutrition, and that all scientific therapeutics consists in re-establishing the normal molecular changes in the material tissues—the principal method whereof is by securing a healthy blood to flow over upon the diseased part—and not simply by inhalations and plasters and specifics, as the manner of too many is and has been since time immemorial. Arsenic is not a specific for asthma or even snake bites.

*Lectures on the Diseases of Women. By Charles West, M. D., Physician Accoucheur to St. Bartholomew's Hospital, etc., Second American from the Second London Edition; Blanchard & Lea*, 1861.—The popularity of this work is well manifested by the brief period required to exhaust the first edition, both in England and this country. Those who read the first part as it appeared in detached portions in the *Medical News and Library*, will well recollect the anxiety to secure the entire work, tantalized only by the monthly morsels. Both parts being now completed and professionally endorsed by rapid passage to a second edition, we need do scarcely more than announce the fact to our readers.

Dr. West writes little, except on those subjects wherein he has enjoyed large practical opportunities for observation, as he well remarks in his preface, that he has "always regarded mere compilation, uncontrolled by large experience, as more apt to perpetuate error than to diffuse truth."

The range of subjects is quite extensive. Commencing with a detail of symptoms and methods of examination, he passes naturally to an investigation of the various disorders of menstruation. Then follows discussion of acute and chronic inflammatory diseases of the uterus and kindred affections. Then all the varied forms of misplacements, including those of the uterus, vagina, rectum and bladder. Next of uterine tumors and outgrowths. Then of malignant or cancerous diseases of the womb. Diseases of the appendages follow in order, including all the forms of ovarian tumors, dropsy, etc. The concluding lectures are devoted to diseases of the bladder, of the urethra and vagina, and lastly of the external organs of generation.

The manner of the author is excellent; his descriptions graphic and perspicuous, and his treatment up to the level of the time—clear, precise, definite and marked by strong common sense.

Those who have read the author's very readable and instructive book on Diseases of Children, would recognize the same gifted mind were not the name on the title page. He is no specialist, no "womb doctor," although every page shows the accomplished medical scholar, observer and practitioner. Messrs. Blanchard & Lea have laid the profession under obligation by this valuable reprint.

---

*Diminutive Products of Labor.*—A correspondent of the *Reporter* chronicles the case of a lady who gave birth to a child which by careful measurement weighed not quite *two pounds.* At the end of a year it weighed fifteen pounds. It is now seven years of age and of average size. A second birth gave a child which weighed two pounds and ten ounces. This child also grew and prospered. Another still more extraordinary case the writer vouches for, although not occurring in his own practice. The child born at full time weighed only a pound and a half. The next child of the same mother weighed ten pounds and a half. Mother and children are still living. The correspondent states that these births occurred in "families of the highest respectability." We may admit the statement, but still insist that the first three cases were "small business."

---

*Puerperal Convulsions treated by Inhalation of Ether.*—Dr. Ayer reports, to the Bost. Soc. for Med. Improvement, two cases of puerperal convulsions successfully treated by anaesthesia produced by ether. The result was in each all that could be desired, relief being prompt and complete. In each case the child was born alive and survived. Thus cases are rapidly accumulating to show that the sanguinary treatment so long depended upon, is wholly unnecessary—beyond this that it is inexcusable in practitioners who are up to the times.

---

*Hæmorrhoids.*—Mr. E. H. Sargent, corner of State and Randolph streets in this city, showed us a little instrument capitally adapted to apply ointments of various sorts to the internal surface in cases of hæmorrhoids. We should judge it also very convenient in some cases of dysentery. It is a little cylinder of hard rubber, with the piston reaching to the end of the tube. The piston being nearly withdrawn the tube is filled with the cerate, and then introduced into the bowel—by pressing upon the piston, as the tube is gradually drawn down, the medicament is freely applied over the diseased surface. Its cleanliness and facility of use render it very eligible. All practitioners know that the ordinary methods which patients use in applying ointments prescribed for this troublesome difficulty, are almost wholly useless. With this simple apparatus they will often prove entirely adequate to the cure.

---

*Congestion of the Brain.*—In looking over the list of deaths in the secular papers one cannot fail to notice the very frequent occurrence of the term *congestion*, in connection with some organ as the cause of death. It will of course be recognized by the pathologist that congestion, even if actually present, is but an incident in the train of phenomena ensuing in death. Determination of blood and stasis must have each a cause or causes. The one being the effect of tissue metamorphosis, at the point of determination, and at the other extremity of the nerve fibres connecting with the terminal arterial twigs transmitting the blood. Analogous physical causes eventuate in stasis or congestion. Subsequent changes, resolution, "passive congestion," modification of nutrition of whatever character, depend upon general or local conditions many of which are well known.

The molecular changes or their cessation, are dependent on local causes, upon the condition of the blood, or upon centric or ex-centric change in the vesicular or conducting neurine. Thus the symptoms of "congestion of the brain" may be presented by depression of an investing bone of the cranium, by apoplectic extravasation, by alcoholic or other stimulants, by narcotics, by secondary organic compounds non-excreted; by

"reflex" nerve influence, &c., &c. Prominently poisons introduced from without, and poisons generated within, carried by the blood to the part or operating less directly through the nervous system. Each and all produce congestion of the brain, as they might produce congestion of any other organ. To understand well the difficulty, the changes induced in the part by nerve conduction must be as definitely before the mind as is the change wrought by the varied constituents of the blood.

Time was when the semi-coma of continued or remittent fevers was baptized congestion, and unfortunately this congestion was understood as implying excessive fullness of the bloodvessels; and bleeding, by at least leeches and cups, was deemed indispensable. Some relics of this rough method infest the present time.

How often the retained urea poisons the blood and produces congestion! How often the typhoid fever poison—how often the pahedal, or, again, the retained elements of bile! Experience has shown, as a more exact pathology inculcated, that often the congestion is to be met by the most powerful stimulants—often by sedatives,—and almost invariably, concurrently or subsequently, by eliminants and nutrients.

---

*Mortality among Medical Journals.*—At the commencement of the present volume, we numbered a goodly array of exchanges. The editorial heart was constantly rejoiced by their familiar faces, but first came the war and the journals of Dixie became contraband, and we mourn for them but not as those without hope, for we steadfastly believe that, when secession is burned to ashes, they will Phœnix like arise therefrom, and on the wings of the *United States* mail return to us again. Then came panic and hard times, and, alas, one half at least of our northern exchanges have been ruthlessly cut down. Here and there the survivors look out to each other with rueful faces—*rari nantes in gurgite vasto*—and we are half afraid to inquire, or even surmise, who next?

A goodly number of the profession have gone to the wars —a multitude which no man can number are seeking to go, for their patients have gone thereunto, and " thither will the eagles be gathered together."

Of one thing rest assured, Beloved Subscriber, war or peace, the CHICAGO MEDICAL JOURNAL will still continue its accustomed visits—so long as there is a shot in the editorial locker, a profession to sustain, and space in the human mind for improvement and advance.

By the way—have you paid up your arrearage? If not make haste, pay what thou owest, and go and sin no more.

---

*The Physician's Pocket Dose and Symptom Book. By Joseph H. Wythes, M. D. Third Edition.* Lindsay & Blakiston, 1861. This is a very convenient little work for students and junior practitioners, containing in a small compass a great deal of valuable information. Many times when waiting upon a tedious case of labor, or from any other cause of delay away from the office and larger books, the student or young practitioner can gleam much of permanent advantage, by conning the pages of such vest pocket compendiums as this. Knowledge never comes without effort, and an immensity can be acquired by saving bits and fragments of time. This little book, therefore, we count valuable, as we do many others that can be slipped in the pocket and then made use of when the moments would otherwise be wasted.

---

*How does Chloroform produce Death.*—H. Culbertson, M. D., of Zanesville, Ohio, in a paper read before the State Medical Society, published in the Cleveland *Gazette*, concludes as the result of a series of experiments on the inferior animals as follows:

1st. That chloroform can not be applied either directly to the brain, or through the medium of the circulation, to an amount proportionate to the powers and idiosyncrasies of the ndividual, without producing *unconsciousness*.

2d. That chloroform to induce insensibility must enter the circulation and locally impress the sensorium.

3d. That anæsthesia may be aided by the presence of carbonaceous compounds in the blood, but that this is subsidiary to the presence of the chloroform in the blood acting upon the nervous centres.

4th. That chloroform diluted as it is by the serum when it enters the blood at the lungs, and onward in its course, can not produce any change in the functional action of the red globules.

5th. That the red globules may be corrugated and deformed by the action of undiluted chloroform out of the vessels, but not when diluted.

6th. That aggregation of the red corpuscles is not a peculiar effect of chloroform on these, but arises from approaching stasis within, and from a well known aggregative tendency when without the vessels.

7th. That chloroform first induces excitement and contraction of the vascular system, and then sedation and dilatation, and ultimately stasis.

---

*Phthisis Contagious.*—"Lector," in the *Lancet & Observer*, urges discussion of the question whether consumption be contagious, and adduces several instances in support of the affirmative. A host of facts can be adduced on both sides. We confess to having been dubious in the past, and at the present, the writer, like the Judges of the Supreme Court of Michigan on the question of the constitutionality of the prohibitory liquor law a few years since, remains equally divided in his own mind. It is a question difficult or impossible of solution, from the simple fact that it is about fruitless to attempt separation of the influence of concurrent cases. This thing is unquestionably true—no person should remain shut up in a room with a consumptive patient, "or any other man," with whatever disease. The seeds of phthisis are easily sown even in what seem the best constitutions. Free ventilation will rob even the most certainly contagious diseases of the major part of their danger, whether to patient or attendant. The evils of pure air even when admitted into the cavities of the joints, but especially to the chambers of the sick, have been vastly overrated. On the other hand there are very few diseases which an impure confined air will not render noxious to attendants, even though a similar one may not be produced. It is not simply the air of the crowded hospital which may become pestilential. To "smell like a sick room," ought to be an obsolete expression, but unfortunately it is not. The sagacious practitioner will see to it that his patient has as pure air as the country affords. And, by the by, John, those bottles and gallipots and pill boxes, and bowls and tumblers with filthy spoons in them, on the table there in sight of the patient, affect his nostrils prodigiously, and he eyes them as a criminal would the branding irons—be kind enough to remove them to the kitchen or stable, and bring me a trifle of eau de Cologne to protect mine own olfactories withal.

---

*Inflamed Mamma.*—Dr. Hale, in the Druggist's Circular, recommends: ℞ Fluid Extract of Aconite ℥ ss; Fl. Extract of Phytolacca, ℥ j; Iodide of Potassium, ʒ j; Warm Water, Oj. Mix. Apply constantly by means of linen compresses.

---

*Chronic Coryza.*—Soubrier's Powder is recommended as an errhine in chronic coryza—ten, twelve or more pinches to be snuffed every day. It is composed of four parts of Subnitrate of Bismuth; eight of powdered liquorice, and thirty of Iodide of Lead, well pulverized and thoroughly mixed.

---

*Chronic Rheumatism.*—Dr. Blackburn of Georgia commends the following electuary in chronic rheumatism and also in some cases of Amenorrhœa: ℞: Pulv. Cinchonae ʒ ij; Pulv. Gum Guaiac. ʒ j; Bi Tart. Potassae ℥ j; Flor. Sulphuris ℥ ss; Pulv. Zingiber ʒ j; Syrup q. s. M. ft. Elect. Of which a teaspoonful or enough to keep the bowels gently open should be given three times a day.

*Chronic Camp Diarrhœa.*—A good old fashioned combination is: ℞ Zinci Sulphat. Solut. Saturat. partes iij. Tr. Opi part. V. M. S. From 15 to 30 drops three times a day.

Another: ℞: Bismuthi Sub. nit. qr. iij—V; Zinci Oxid.; qr. i–ij; Ext. Opi qr. ¼–j. M. S. This to be taken from three to six times a day.

Very often when chalybeates are indicated the Liq. Ferri Persequiseit; in full doses alone or combined with small doses of Strychnia. Several apparently uncontrollable cases have speedily yielded to moderately large doses of Fowler's Solution guarded by a few drops of Tr. Opi. One case which resisted astringents and alteratives of all sorts, where the diet was regulated with the greatest precision and the most careful hygiene enforced and all without benefit, was cured almost immediately by plentiful imbibition of simple Liquor Calcis.

One of our subscribers, a Brigade Surgeon, promises us an article on the cure of camp diarrhœa by free employment of Veratrum Viride and Acetate of Lead. (It is with feelings of extreme surprise that we have not yet to chronicle the magical effects of Chlorate of Potash in this difficulty. Will the light of this panacea not shine along the uneasy bowels of the soldiery?) Equal parts of Acid. Sulph. Aromat. and McMunn's Elixir Opi, in thirty drop doses, three times a day, is recommended by an Army Surgeon in whom we have high confidence.

A great variety of remedial agents will be found convenient, if not indispensable.

---

*A Treatise on Diseases of the Joints. By Richard Barwell, F. R. C. S., Assistant Surgeon Charing Cross Hospital, &c. Blanchard & Lea; Philadelphia,* 1861. *8vo., pp.* 463. *From the Publishers.*

This is a very valuable work, treating as it does of a class of diseases of vast importance. Few treatises on the subject are worthy of note since Brodie's well known work, and the advances in Physiology, Pathology and Therapeutics since that was given to the profession have been so great, that the time demanded renewed posting up of the whole matter.

A glance at the order of topics discussed by the writer will sufficiently commend the work to the reader's notice, when he is assured that the author is amply qualified for his task.

Ch. I—Physiological Anatomy of the Joints; Ch. II—Acute Synovitis; Ch. III—Acute Rheumatism; Ch. IV—Pyarthrosis; Ch. V—Stumous Synovitis; Ch. VI—Rheumatic Synovitis; Ch. VII—Other forms of Chronic Synovitis; Ch. VIII—Hydarthosis; Ch. IX—Loose Cartilages in the Joints; Ch. X—Acute Articular Osteitis; Ch. XI—Strumous *do.;* Ch. XII—Chronic Rheumatic Arthritis (Osteitis); Ch. XIII—Inflammation and Degluration of Cartilages; Ch. XIV—Hipjoint Disease; Ch. XV—Affections of Synovial Sheaths and Bursæ; Ch. XVI—Hysteric Pseudo Disease of the Joints; Ch. XVII—Restoration of Mobility and Conformity of Crippled Joints; Ch. XVIII—Removal of Diseased Joints.

This is a bill of fare worthy a professional epicure, and the author is clear, explicit and up to the times in his classification, pathology and diagnosis. The chapter on Pyarthrosis will attract large attention, particularly wherein he alludes to the uterine and gonorrhœal source of pus. The latter proposition will meet considerable discussion, before reception, by many minds.

It is to be regretted that the author's therapeutics are by no means up to the level of his pathology and diagnosis. The local treatment is unworthy the association it has too often found. Another capital fault is the omission in large part of the various mechanical appliances, better known in this country than Europe, but recognized by many of the best surgeons even there, which have so immensely advanced successful treatment of diseased joints in this time and this country. But, aside from these defects, the merits of the work must ensure it a cordial reception by all reading practitioners.

*Nourishment of the Wounded in Battle.*—As soon as the battle commences beef-tea should be prepared in large quantities, by cutting up the beef of the horses which have been killed, in lieu of ox-beef, if that is absent or scarce, in a barrel, and adding hydrochloric acid and water. The wounded, in a painful journey to the rear, and awaiting operation, will be wonderfully sustained and refreshed by this fluid nutriment, and the horrible thirst of the wounded, which is but acute hunger, will be effectually and most advantageously appeased. The mortality, after field operations of even the gravest character, will be diminished in a remarkable manner if sufficient support and nutrition are supplied between the two points of injury and operation, and the reception into general hospitals. If the veins are supplied with healthy nutrition, they will have less temption to absorb pus.—*Amer. Medical Times.*

*Dupuytren on Spurious Pregnancy.*—Prof. Simpson, in lecturing on spurious pregnancy, tells the following good story of Dupuytren: "Such cases are, happily, extremely rare; but you do meet with them occasionally, and not in patients within the walls of a lunatic asylum only. It is told that a lady once came to Dupuytren, to ask what was to be done in her case, as she had now been in a family way for fourteen years—and the great Parisian surgeon gave it as his opinion, that as the boy must be tolerably well grown by that time, the best thing the lady could do was to swallow a tutor immediately, that his education might not be neglected."—*Medical News and Library.*

*Sylvester Method of Restoring Suspended Animation.*—This method, which has supplanted with many the Marshall Hall method, is thus described:

"Dr. Sylvester's method is to lay the patient on his back, and having pulled the tongue forward, to draw the arms slowly up over the head, by which means the ribs are elevated by the pectoral muscles, and inspiration is produced; the arms are then to be brought down to the side of the chest, which they are to compress in a slight degree. These movements are to be repeated as slowly as in the Marshall Hall method, and it is said that they give a more complete change of air to the lungs."

